# Breaking barriers: harnessing hypofractionated radiotherapy to transform outcomes in low tumor mutation burden stage III non-small cell lung cancer - a retrospective study

**DOI:** 10.3389/fimmu.2025.1557154

**Published:** 2025-04-09

**Authors:** Jingyun Yang, Tianxiang Cui, Yang Zhang, Guangpeng Chen, Xinxin Wang, Jianguo Sun, Anmei Zhang, Guanghui Li

**Affiliations:** Institute of Cancer, Xinqiao Hospital, Army Medical University (Third Military Medical University), Chongqing, China

**Keywords:** non-small cell lung cancer, hypofractionated radiotherapy, conventional fractionated radiotherapy, tumor mutation burden, progression-free survival

## Abstract

**Background:**

Non-Small Cell Lung Cancer (NSCLC) patients with low tumor mutational burden (TMB) showed low sensitive to conventional fractionated radiotherapy in our previous study. This study aimed to evaluate the efficacy and safety of hypofractionated radiotherapy (HFRT) in locally advanced NSCLC patients with low-TMB compared to conventional fractionated radiotherapy (CFRT).

**Methods:**

We retrospectively analyzed clinical outcomes of 74 locally advanced NSCLC patients with low-TMB undergoing definitive radiotherapy from January 2017 to July 2023, with 31 patients received HFRT (received radiation doses of >2Gy and ≤5 Gy per fraction) and 43 received CFRT (received radiation doses of 1.8-2 Gy per fraction). Progression-free survival (PFS), overall survival (OS) and objective response rate (ORR) to radiotherapy was analyzed in the two groups. Univariate analysis was performed to assess the impact of clinical characteristics on PFS. We also analyzed PFS in subgroups receiving HFRT or CFRT combined with immunotherapy and chemotherapy.

**Results:**

Survival analysis revealed the median PFS of 13 months in the HFRT group was significantly better than the 10 months in the CFRT group (p = 0.024). The 6-month and 12-month PFS rates were 80.6% and 61.3% for the HFRT group, versus 81.4% and 39.5% for the CFRT group, respectively. Median OS was 27 months in the HFRT group and 20 months in the CFRT group (p = 0.079). There were no statistically significant differences in major adverse events between the HFRT and CFRT groups (all p>0.05). In the subgroup receiving combined immunotherapy and chemotherapy, the median PFS was 10 months in the HFRT group and 9 months in the CFRT group (p = 0.092).

**Conclusion:**

HFRT was superior to CFRT in prolonging PFS for patients with low-TMB locally advanced NSCLC. It was a safely and effective approach for these patients and was worth further prospective studies with larger sample sizes.

## Introduction

Lung cancer is now the leading malignancy in both incidence and mortality worldwide (1). Over the past few decades, its global incidence has increased dramatically, with rates rising 3.8-fold in women and 10.3-fold in men. This trend poses a significant threat to public health (2). Non-small cell lung cancer (NSCLC) accounts for about 80% of all lung cancer cases. In China, over 75% of NSCLC patients are diagnosed at an advanced stage, rendering them ineligible for surgery. These patients tend to have poor outcomes, with high mortality rates and an overall 5-year survival rate of less than 20% (3, 4). For locally advanced NSCLC, concurrent chemoradiotherapy is the standard treatment (5). However, 40-50% of patients still experience local failure or relapse after conventional radiotherapy, largely due to radioresistance (6). Increasing the radiotherapy dose could improve local control, but this approach is limited by the tolerance of surrounding normal tissues. Currently, the uniform radiotherapy dose does not consider variations in radiosensitivity among individual tumors (7, 8). Understanding individual radiosensitivity is crucial. Implementing personalized radiotherapy could improve both survival outcomes and quality of life for those with locally advanced NSCLC.

Our previous analysis of The Cancer Genome Atlas (TCGA) database showed a positive correlation between TMB and survival in NSCLC patients who had undergone radiotherapy (9). Our work revealed that radiosensitivity in NSCLC can be stratified based on TMB levels. Patients with low TMB are less responsive to both radiotherapy and immunotherapy, representing a significant treatment challenge. Recent research has focused on optimizing fractionation schedules to increase the biologically effective dose (BED). The CALGB 31102 phase I trial indicated good tolerance for the hypofractionated regimen (10). Kaster et al. (11) summarized 33 studies on hypofractionated radiotherapy in stage III lung cancer patients from 1990 to 2014, showing a positive correlation between the 5-year survival rate and the BED. For every 1 Gy increase in BED, the 5-year survival rate increased by 0.36%-0.70%. Currently, hypofractionated radiotherapy for locally advanced NSCLC uses a per-fraction dose slightly higher than conventional schemes, defined as >2 Gy per fraction. This “moderate” hypofractionation approach not only shortens the overall treatment duration and limits tumor repopulation but also avoids severe late radiation toxicity caused by overly high doses per fraction (12). This radiotherapy modality might serve as an effective approach to improve the prognosis of locally advanced NSCLC patients with low-TMB.

This study retrospectively analyzed the efficacy and safety of hypofractionated radiotherapy versus conventional fractionated radiotherapy in locally advanced NSCLC patients with low-TMB, laying the groundwork for future phase III randomized controlled trials.

## Methods

### Patient eligibility

We retrospectively collected efficacy and safety data from patients with stage III NSCLC who received definitive radiotherapy at the Department of Oncology, Second Affiliated Hospital of Army Medical University, between January 2017 and July 2023. The staging of these patients was determined according to the eighth edition of the American Joint Committee on Cancer (AJCC) guidelines, using chest and abdominal CT/abdominal ultrasound, brain MRI, bone scan, or whole-body PET-CT.

Patients included in this study met the following criteria: (1) age ≥ 18 years with stage III NSCLC confirmed by histopathology, deemed inoperable, declined surgery, or unsuitable for surgery due to underlying conditions; (2) all patients underwent genetic testing, with TMB ≤16 Muts/Mb (13); (3) CFRT group: received radiation doses of 1.8-2 Gy per fraction, HFRT group: received radiation doses of >2Gy and ≤5 Gy per fraction; (4) Eastern Cooperative Oncology Group (ECOG) performance status (PS) of 0-2. Patients previously treated with definitive radiotherapy, or those with other malignancies or severe comorbidities likely to affect survival, were excluded from the study.

The included patients were divided into two groups based on the radiotherapy fractionation schedule: the conventional fractionation group (dose per fraction 1.8-2 Gy) and the hypofractionation group (dose per fraction >2 Gy). The registry followed ethical guidelines for epidemiological research, and the study protocol was approved by the Institutional Review Board of the Second Affiliated Hospital of Army Medical University. In line with ethical standards for retrospective analyses, all patient data were anonymized to ensure privacy.

### Data collection and response evaluation

Patient data extracted from the system included age, sex, smoking status, ECOG PS, pathology, genetic testing results, clinical stage at the start of radiotherapy, radiotherapy dose, radiotherapy-related side effects, systemic therapies administered within 6 months before or after radiotherapy, and the time from the start of radiotherapy to the first progression of the disease. Progression-free survival (PFS), overall survival (OS), and tumor response were evaluated. Tumor response was assessed according to version 1.1 of the Response Evaluation Criteria in Solid Tumors (RECIST).

### Statistical analyses

PFS and OS were analyzed using the survminer package in Windows R version 4.2.2, and survival curves were created with the Kaplan-Meier method. Group differences in survival rates were evaluated using the log-rank test. Descriptive statistics were generated with IBM SPSS Statistics version 27 for Windows, and comparisons were made using the chi-square test, with additional validation through Fisher’s exact test. Univariate analysis relied on the Kaplan-Meier method. All p-values were two-sided, and a p-value < 0.05 was considered statistically significant.

## Results

### Cohort characteristics and treatment

Between January 2017 and July 2023, a total of 74 patients were included in this study(screened from a total of 718 cases), with a median age of 58 years (range 42-76 years). Among them, 30 patients (40.5%) were aged ≥ 60 years. The cohort included 54 males (73.0%) and 20 females (27.0%). Thirty-three patients (44.6%) had a smoking index ≥ 400 pack-years. Pathological types included adenocarcinoma (52.7%) and squamous cell carcinoma (47.3%). Most patients had a PS of 1, with 63 patients (85.1%) classified as PS 1 and 11 patients (14.9%) as PS 2. Regarding tumor staging, 41 patients (55.4%) were stage IIIA, 25 patients (33.8%) were stage IIIB, and 8 patients (10.8%) were stage IIIC. Genetic testing was performed in all patients, revealing high PD-L1 expression (≥1%) in 22 patients and low PD-L1 expression (<1%) in 52 patients. Additionally, 31 patients (41.9%) received hypofractionated radiotherapy (HFRT), while 43 patients (58.1%) received conventional fractionated radiotherapy (CFRT). All patients underwent systemic therapies administered within 6 months before or after radiotherapy: 11 patients (35.5%) from the HFRT group and 12 patients (27.9%) from the CFRT group received chemotherapy, while 20 patients (64.5%) in the HFRT group and 31 patients (72.1%) in the CFRT group received both chemotherapy and immunotherapy. In summary, the baseline characteristics well balanced except for the smoking index. Detailed patient characteristics are shown in **Table 1**.

**Table 1:** Baseline participant characteristics in the overall patient cohort.

Variable	N		P value
	HFRT(n=31)	CFRT(n=43)	
Age-yr (%)			0.337
Md(range)	59 (42-72)	57 (44-76)	
≥ 60	15 (48.4)	15 (34.9)	
<60	16 (51.6)	28 (65.1)	
Gender (%)			0.434
Male	21 (67.7)	33 (76.7)	
Female	10 (32.3)	10 (23.3)	
Smoking Index (%)			0.033
≥400 cigs/yr	9 (29.0)	24 (55.8)	
<400 cigs/yr	22 (71.0)	19 (44.2)	
Pathology Type (%)			0.485
Adenocarcinoma	18 (58.1)	21 (48.8)	
Squamous Cell Carcinoma	13 (41.9)	22 (51.2)	
PS (%)			0.340
0-1	28 (90.3)	35 (81.4)	
2	3 (9.7)	8 (18.6)	
TNM stage (%)			0.266
IIIA	19 (61.3)	22 (51.2)	
IIIB- IIIC	12 (38.7)	21 (48.8)	
PD-L1 Expression Levels (%)			0.798
High Expression (≥1%)	10 (32.3)	12 (27.9)	
Low Expression (<1%)	21 (67.7)	31 (72.1)	
Types of Systemic Therapies (%)			0.612
Chemotherapy	11 (35.5)	12 (27.9)	
Chemotherapy + Immunotherapy	20 (64.5)	31 (72.1)	
Chemotherapy Timing (%)			0.802
Concurrent Chemotherapy	11 (35.5)	13 (30.2)	
Sequential Chemotherapy	20 (64.5)	30 (69.8)	

### Response and survival

**Figure 1:**
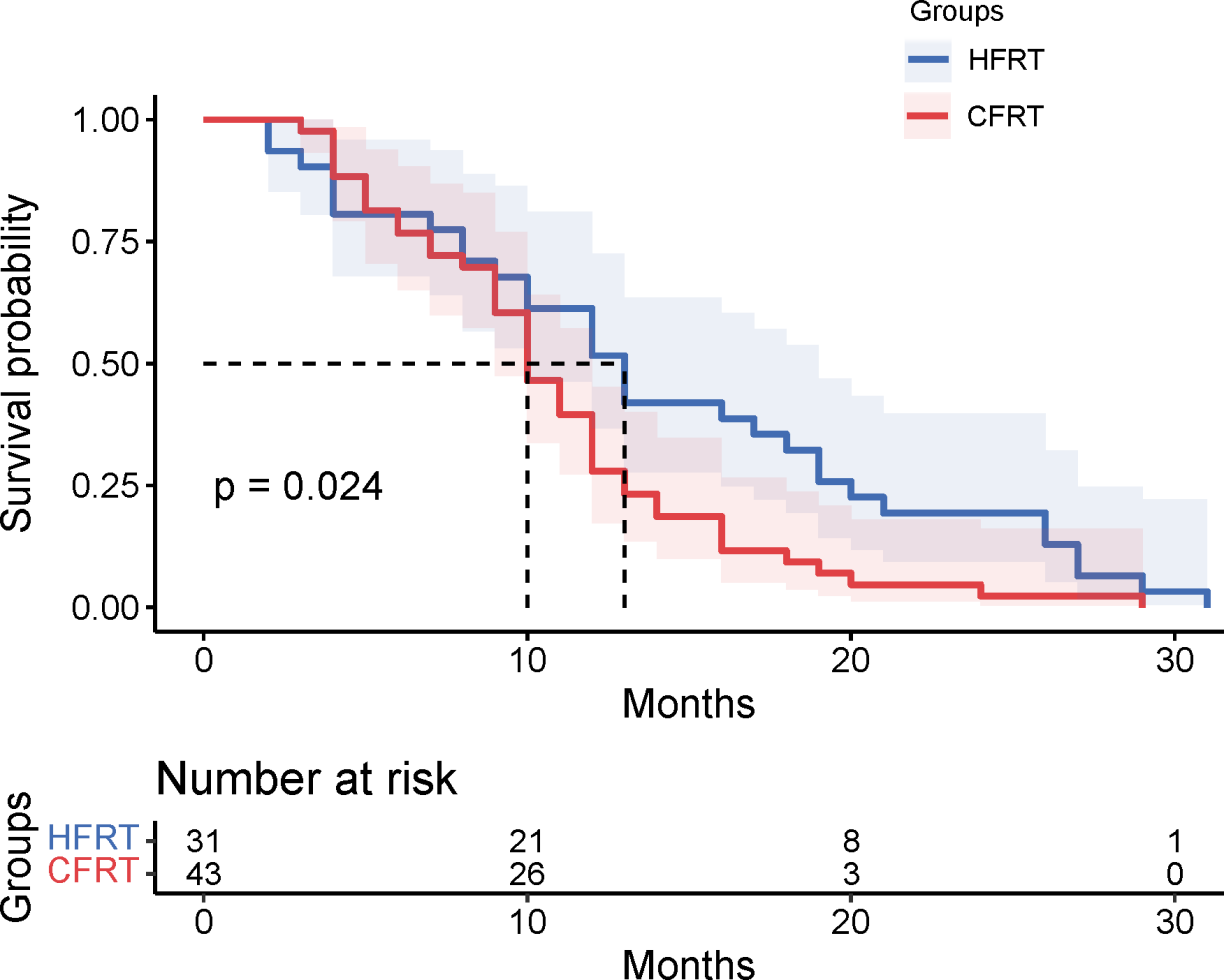
Kaplan-Meier survival curves for PFS in the HFRT and CFRT groups. The median PFS was significantly longer in the HFRT group compared to the CFRT group (P=0.024). Shaded areas represent 95% confidence intervals. The table below indicates the number of patients at risk at various time points. PFS, progression-free survival;HFRT, hypofractionated radiotherapy; CFRT, conventional fractionated radiotherapy.

**Figure 2:**
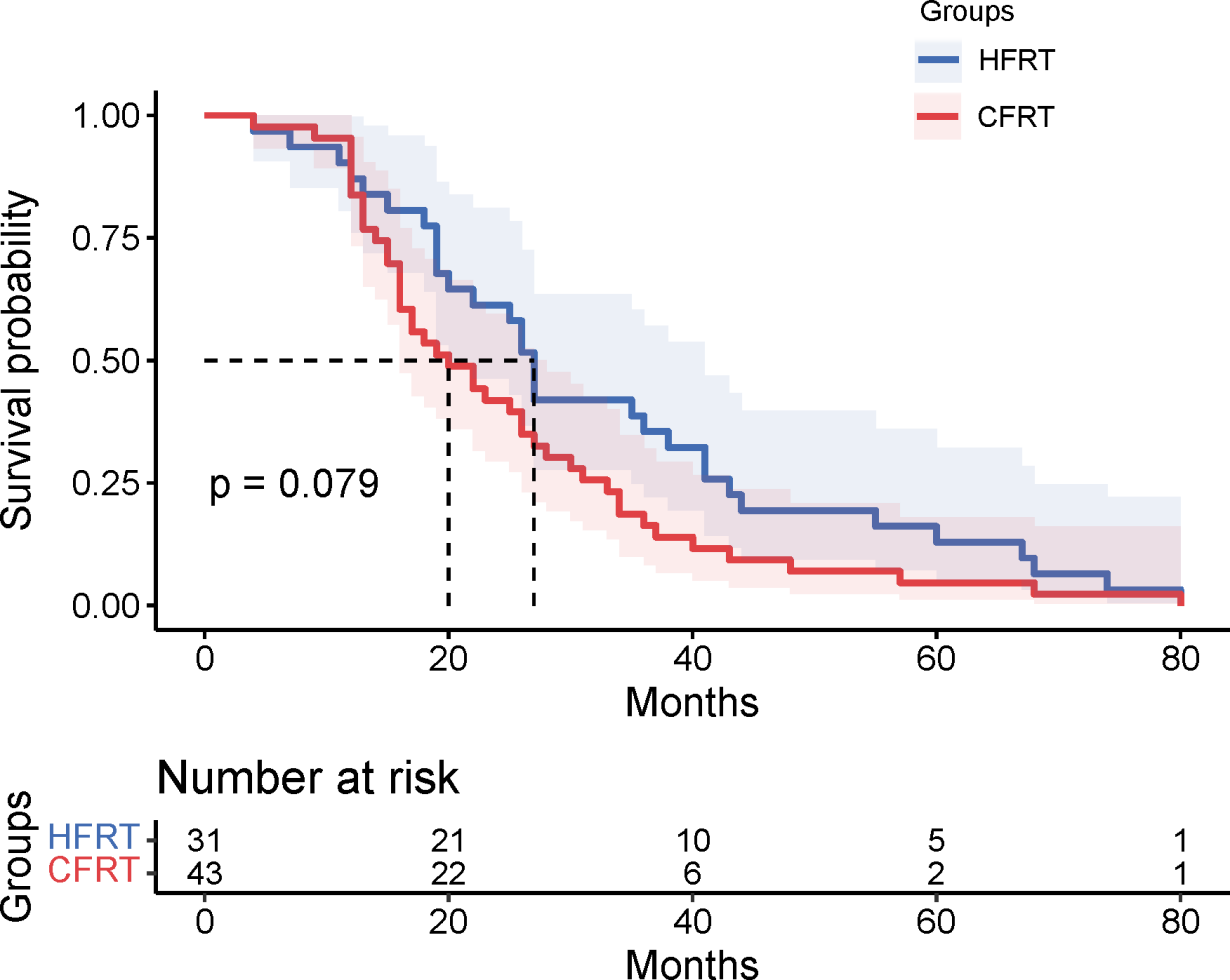
Kaplan-Meier survival curves for OS in the HFRT and CFRT groups. The median OS was 20 months for the HFRT group and 15 months for the CFRT group, with no statistically significant difference observed (P=0.079). Shaded areas represent 95% confidence intervals. The table below indicates the number of patients at risk at various time points. OS , overall survival; HFRT, hypofractionated radiotherapy; CFRT, conventional fractionated radiotherapy.

**Figure 3:**
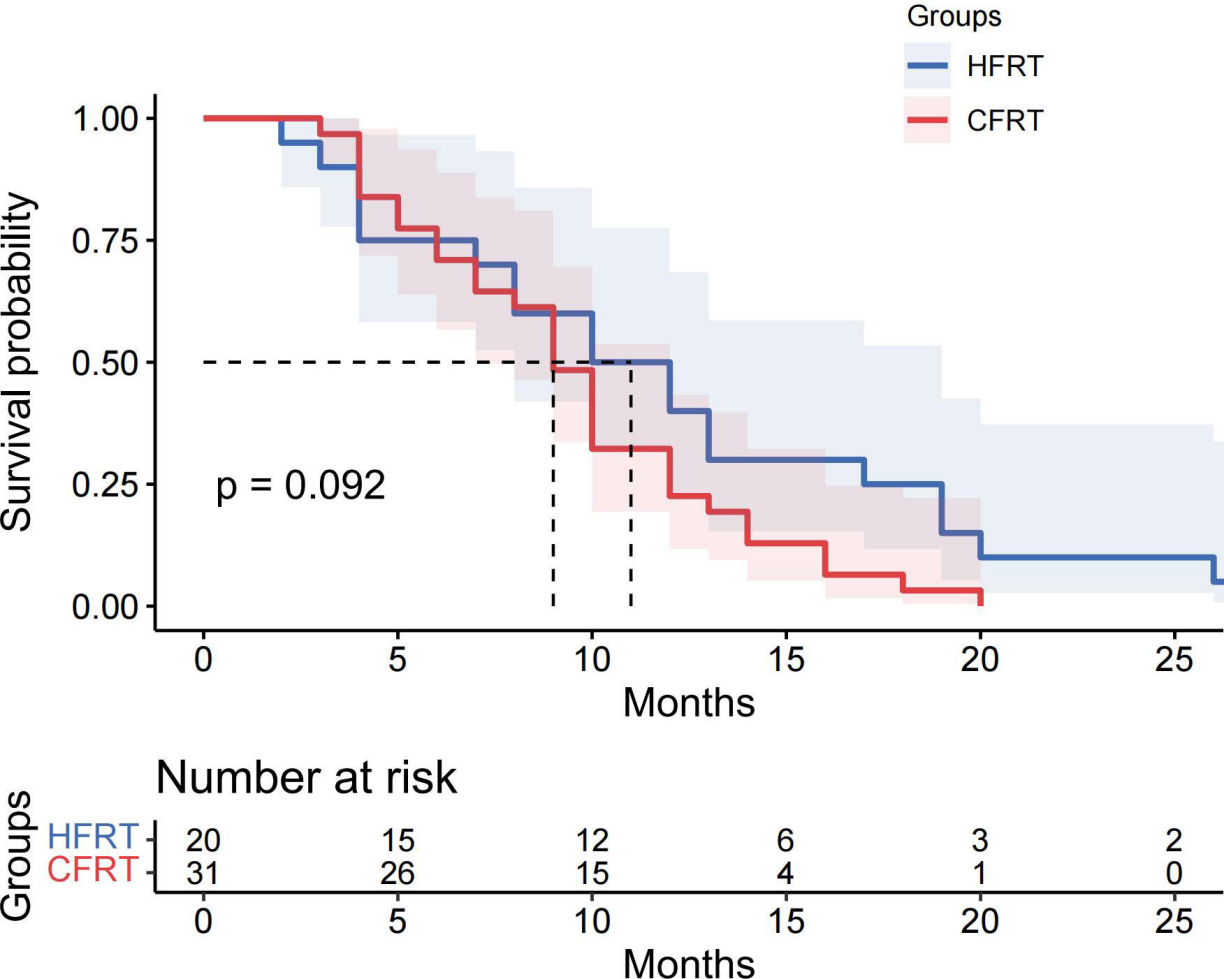
The Kaplan-Meier survival curves for PFS in the subgroup receiving combined immunotherapy and chemotherapy with radiotherapy. The median PFS was 10 months for the HFRT group and 9 months for the CFRT group, with no statistically significant difference observed (P=0.092). Shaded areas represent 95% confidence intervals. The table below indicates the number of patients at risk at various time points. PFS, progression-free survival; HFRT, hypofractionated radiotherapy; CFRT, conventional fractionated radiotherapy.

This study evaluated the differences in survival outcomes and treatment efficacy between HFRT and CFRT. Survival analysis revealed that the median PFS was 13 months in the HFRT group, which was significantly better than the 10 months in the CFRT group (P = 0.024). The 6-month and 12-month PFS rates were 80.6% and 61.3% for the HFRT group, compared to 81.4% and 39.5% for the CFRT group, suggesting that HFRT is superior to CFRT in delaying disease progression (**Figure 1**). The median OS was 27 months in the HFRT group and 20 months in the CFRT group, with no significant difference between the two groups (P = 0.079), although the HFRT group showed a trend toward improved survival (**Figure 2**). In the subgroup receiving combined immunotherapy and chemotherapy, the median PFS was 10 months in the HFRT group and 9 months in the CFRT group (p = 0.092), although the HFRT group showed a trend toward improved PFS (**Figure 3**). although it did not reach statistical significance.

Regarding radiotherapy response, the objective response rate (ORR) in the HFRT group was 32.3%, compared to 23.3% in the CFRT group, with no statistically significant difference observed (P = 0.434). The disease control rate (DCR) was 90.3% in the HFRT group and 95.3% in the CFRT group, similarly showing no statistical significance (P = 0.644) (**Table 2**).

**Table 2:** Comparison of treatment response between HFRT and CFRT groups.

Variable	HFRT (%)	CFRT (%)	P value
No of patients	31	43	
Overall response	31	43	
Complete response	0	0	
Partial response	10 (32.3)	10 (23.3)	
Stable disease	18 (58.1)	31 (72.1)	
Progressive disease	3 (9.6)	2 (4.6)	
ORR	10 (32.3)	10 (23.3)	0.434
DCR	28 (90.3)	41 (95.3)	0.644

HFRT, hypofractionated radiotherapy; CFRT, conventional fractionated radiotherapy.

### Safety

**Table 3** shows the main adverse events in patients who received HFRT and CFRT. Grade 2 or higher esophagitis occurred in 2 patients in each group (HFRT: 6.5%, CFRT: 4.7%, P = 1.000). Grade 3 or higher pneumonitis was reported in 6 patients from the HFRT group (19.4%) and 9 patients from the CFRT group (20.9%, P = 1.000). Grade 3 or higher leukopenia was found in 2 patients (6.5%) in the HFRT group and 4 patients (9.3%) in the CFRT group (P = 1.000). Grade 3 or higher thrombocytopenia was observed in 3 patients (9.7%) in the HFRT group and 2 patients (4.7%) in the CFRT group (P = 0.644). Only 1 patient in the CFRT group experienced ≥ grade 3 anemia (2.3%, P = 1.000). Overall, severe (≥ grade 3) adverse events were rare during radiotherapy. HFRT did not increase radiotherapy-related toxicities compared to CFRT in this study.

**Table 3:** Comparison of adverse events between HFRT and CFRT groups.

Adverse Events	Grade	HFRT (%)	CFRT (%)	P value
Esophagitis	≥2	2 (6.5)	2 (4.7)	1.000
	0-1	29 (93.5)	41 (95.3)	
Pneumonia	≥3	6 (19.4)	9 (20.9)	1.000
	0-2	25 (80.6)	34 (79.1)	
Leukopenia	≥3	2 (6.5)	4 (9.3)	1.000
	0-2	29 (93.5)	39 (90.7)	
Thrombocytopenia	≥3	3 (9.7)	2 (4.7)	0.644
	0-2	28 (90.3)	41 (95.3)	
Anemia	≥3	0 (0)	1 (2.3)	1.000
	0-2	31 (100)	42 (97.7)	

HFRT, hypofractionated radiotherapy; CFRT, conventional fractionated radiotherapy.

### Factors affecting progression-free survival (PFS)

In this study, we found that the HFRT group demonstrated better efficacy in terms of PFS. Therefore, we subsequently evaluated the impact of various clinical characteristics on PFS, but no statistically significant differences were observed. The median PFS was similar across different variables. For age, both patients aged ≥ 60 years and those aged < 60 years had a median PFS of 11 months (P = 0.929). Regarding gender, the median PFS was 10 months for male and 12 months for female (P = 0.648). Smoking index also showed no significant effect on PFS, both patients with ≥ 400 pack-years and those with < 400 pack-years having a median PFS of 11 months (P = 0.989). In terms of pathological type, adenocarcinoma patients had a median PFS of 12 months, while squamous cell carcinoma patients had a median PFS of 11 months, with no statistically significant difference (P = 0.134). For PS, patients with PS 0-1 had a median PFS of 11 months, while those with PS 2 had a median PFS of 12 months (P = 0.564). TNM staging also showed no significant differences, with both stage IIIA and stage IIIB-IIIC patients having a median PFS of 11 months (P = 0.090) (**Figure 1** and **Table 4**). Since our findings indicated no statistical significance on OS between HFRT and CFRT, we did not analyze the impact of various clinical characteristics on OS in this context.

**Table 4:** Analysis of clinical characteristics’ impact on progression-free survival (PFS).

Idex	Median PFS (month)	P value
Age (yr)		0.929
≥ 60	11	
<60	11	
Gender		0.648
Male	10	
Female	12	
Smoking Index		0.989
≥400 cigs/yr	11	
<400 cigs/yr	11	
Pathology Type		0.134
Adenocarcinoma	12	
Squamous Cell Carcinoma	11	
PS		0.564
0-1	11	
≥2	12	
TNM stage		0.090
IIIA	11	
IIIB-IIIC	11	

PFS, progression-free survival.

## Discussion

Approximately 20-30% of newly diagnosed NSCLC patients have stage III disease. Stage III NSCLC is a highly heterogeneous group of cancers, characterized by significant variability in tumor size and lymph node involvement. Patients with stage III NSCLC also vary widely in age and comorbidities, further increasing the complexity and challenges of treatment. The standard treatment for stage III NSCLC is concurrent chemoradiotherapy (CCRT) followed by one year of durvalumab maintenance therapy, which has been shown to provide significant survival benefits (14). However, our previous research found that NSCLC patients with low-TMB exhibited reduced sensitivity to both radiotherapy and immunotherapy. For this subgroup, we believe that targeted escalation of the biological dose in radiotherapy could be beneficial. It may improve tumor control rates and enhance sensitivity to immunotherapy. In this study, we sought to achieve a higher biological dose by modifying the fractionation schedule. The therapeutic effectiveness of HFRT (dose > 2 Gy per fraction) was compared with CFRT in locally advanced NSCLC patients with low-TMB.

In fact, preclinical and clinical studies have demonstrated that the doubling time of most cancer cells is less than one week (15, 16). Prolonging the overall treatment duration may lead to tumor repopulation and accelerated growth (16, 17). HFRT may be an effective treatment option, as it can overcome radiation resistance by reducing the total treatment time (18). Previous studies have demonstrated that hypofractionated radiotherapy could be a promising alternative to conventional treatment, with interesting clinical outcomes. For example, Robinson et al. reported a median OS of 22.5 months in the hypofractionation group, with 48% of patients surviving at two years (19). Similarly, Agolli et al. found favorable survival results in their single-institution retrospective series, reporting two-year OS and PFS rates of 40% and 33.5%, respectively, for stage III NSCLC patients (20). However, it remains unclear whether the use of hypofractionated radiotherapy can provide survival benefits for the subgroup of patients with low-TMB, who are generally resistant to radiotherapy. It has been established through research that deleterious DNA damage response and repair (DDR) mutations are frequently observed in NSCLC and are correlated with the heightened levels of tumor-infiltrating lymphocytes, the enhanced genomic instability, and the elevated tumor mutation burden TMB within cancer (21). Higher single-fraction radiotherapy doses can induce more DNA double-strand breaks, leading to apoptosis or even necrosis, while minimizing sublethal damage repair. Our study demonstrated that in the low-TMB subgroup, the median PFS was 13 months in the HFRT group, significantly longer than the 10 months observed in the CFRT group (P=0.024). This finding suggests that HFRT can significantly extend PFS in this subgroup. Although HFRT showed a marked advantage in PFS, the difference in OS between the HFRT and CFRT groups did not reach statistical significance (P=0.079), likely due to the limited sample size of this study. Furthermore, the treatment for NSCLC patients throughout the disease course typically involves multimodal approaches, including chemotherapy, targeted therapy, and immunotherapy. The effects of these combined treatments may partially obscure the impact of radiotherapy on OS. These findings indicate that while HFRT offers a clear advantage in extending PFS for locally advanced NSCLC patients with low -TMB, its ultimate effect on OS requires further validation through longer follow-up and larger-scale clinical trials.

The combination of immunotherapy and radiotherapy is a prominent area of clinical research. Historically, radiotherapy has been used mainly for local disease control, however, recent advances in accurate, high-dose ionizing radiation (IR) delivery have not only improved local tumor control but, in some cases, reduced metastatic burden. These “abscopal” effects of IR on non-irradiated tumor sites are thought to be mediated by T cells triggered by tumor antigens, which migrate to metastatic sites and contribute to tumor regression (22–25). Moreover, combining IR with immune checkpoint inhibitors, such as ipilimumab (anti-CTLA-4) and nivolumab (anti-PD-1), has shown to yield favorable treatment responses (26–28). These studies suggest that local radiotherapy induces changes in tumor immune microenvironment, the release of new antigens, or cell death, which may interact with immunotherapy. Thus, radiotherapy’s ability to enhance immune responses provides a promising synergistic approach with potential substantial clinical benefits. Previous studies have reported that the low-TMB subgroup is less responsive to immunotherapy, but it is worth investigating whether hypofractionated radiotherapy could induce more tumor-related antigens and neoantigens during tumor cell apoptosis and necrosis, thereby enhancing antitumor immune effects. In our study, we evaluated patients who received combined immunotherapy and chemotherapy administered within 6 months before or after radiotherapy. The results showed a median PFS of 10 months in the HFRT group and 9 months in the CFRT group (p = 0.092). Although no significant difference was observed between the two groups, a trend toward prolonged PFS with HFRT in low TMB patients was noted, which may be attributed to the limited sample size. To further investigate whether HFRT can synergistically enhance the efficacy of immunotherapy in locally advanced NSCLC patients with low TMB, future prospective clinical cohort study with large sample size is warranted.

In the baseline assessment of our study cohort, we found a significant difference in the smoking index between the HFRT and CFRT groups (P = 0.033). However, univariate analysis showed that the smoking index did not affect PFS (P = 0.989). We believe this factor does not impact our overall findings. Our study does have some limitations: the sample size was small, and the follow-up period was limited. Additionally, patients received different types of treatments, which might affect the generalizability and statistical power of our results. Despite these limitations, our study provides valuable insights into the use of HFRT for locally advanced NSCLC patients with low-TMB. HFRT showed significant benefits in extending PFS in this particular subgroup. Future research should build on these preliminary findings. We need more rigorous, prospective, multicenter studies with larger sample sizes to validate the effects of HFRT on PFS, OS, and quality of life. Future studies should also explore combining radiotherapy with other treatments such as chemotherapy, immunotherapy, and targeted therapy, and evaluate how these combinations impact PFS and OS. Multimodal treatment has become the standard approach for NSCLC, but the role of radiotherapy within this framework still needs further exploration. For patients with locally advanced NSCLC, the focus should be on individualized treatment to maximize PFS and OS while improving quality of life.

In conclusion, this retrospective analysis demonstrated that for locally advanced NSCLC patients with low-TMB, HFRT had a significant advantage over CFRT in prolonging PFS. This finding provides a new reference for treating patients for locally advanced NSCLC with low-TMB. Patients with low -TMB are generally less responsive to radiotherapy and immunotherapy, but targeted escalation of the BED of radiotherapy can effectively extend PFS in this subgroup. Further large-scale prospective studies are needed to validate these findings.

## Data Availability

The original contributions presented in the study are included in the article/Supplementary Material. Further inquiries can be directed to the corresponding authors.

## References

[1] Siegel RL Giaquinto AN Jemal A. Cancer statistics, 2024. CA Cancer J Clin. (2024) 74:12–49. doi: 10.3322/caac.21820 38230766

[2] Bray F Laversanne M Sung H Ferlay J Siegel RL Soerjomataram I. Global cancer statistics 2022: GLOBOCAN estimates of incidence and mortality worldwide for 36 cancers in 185 countries. CA Cancer J Clin. (2024) 74:229–63. doi: 10.3322/caac.21834 38572751

[3] Chen P Liu Y Wen Y Zhou C. Non-small cell lung cancer in China. Cancer Commun (Lond). (2022) 42:937–70. doi: 10.1002/cac2.12359 PMC9558689 36075878

[4] Blandin KS Crosbie PA Balata H Chudziak J Hussell T Dive C. Progress and prospects of early detection in lung cancer. Open Biol. (2017) 7(9):170070. doi: 10.1098/rsob.170070 28878044 PMC5627048

[5] Auperin A Le Pechoux C Rolland E Curran WJ Furuse K Fournel P. Meta-analysis of concomitant versus sequential radiochemotherapy in locally advanced non-small-cell lung cancer. J Clin Oncol. (2010) 28:2181–90. doi: 10.1200/JCO.2009.26.2543 20351327

[6] Pollom EL Qian Y Durkee BY von Eyben R Maxim PG Shultz DB. Hypofractionated intensity-modulated radiotherapy for patients with non-small-cell lung cancer. Clin Lung Cancer. (2016) 17:588–94. doi: 10.1016/j.cllc.2016.05.024 27378172

[7] Bischoff P Altmeyer A Dumont F. Radiosensitising agents for the radiotherapy of cancer: advances in traditional and hypoxia targeted radiosensitisers. Expert Opin Ther Pat. (2009) 19:643–62. doi: 10.1517/13543770902824172 19441939

[8] Impicciatore G Sancilio S Miscia S Di Pietro R. Nutlins and ionizing radiation in cancer therapy. Curr Pharm Des. (2010) 16:1427–42. doi: 10.2174/138161210791033932 20166982

[9] Jia Q Chu Q Zhang A Yu J Liu F Qian K. Mutational burden and chromosomal aneuploidy synergistically predict survival from radiotherapy in non-small cell lung cancer. Commun Biol. (2021) 4:131. doi: 10.1038/s42003-021-01657-6 33514859 PMC7846582

[10] Urbanic JJ Wang X Bogart JA Stinchcombe TE Hodgson L Schild SE. Phase 1 study of accelerated hypofractionated radiation therapy with concurrent chemotherapy for stage III non-small cell lung cancer: CALGB 31102 (Alliance). Int J Radiat Oncol Biol Phys. (2018) 101:177–85. doi: 10.1016/j.ijrobp.2018.01.046 PMC6173195 29487024

[11] Kaster TS Yaremko B Palma DA Rodrigues GB. Radical-intent hypofractionated radiotherapy for locally advanced non-small-cell lung cancer: a systematic review of the literature. Clin Lung Cancer. (2015) 16:71–9. doi: 10.1016/j.cllc.2014.08.002 25450876

[12] Brand DH Kirby AM Yarnold JR Somaiah N. How low can you go? The radiobiology of hypofractionation. Clin Oncol (R Coll Radiol). (2022) 34:280–87. doi: 10.1016/j.clon.2022.02.009 35260319

[13] Friedman CF Hainsworth JD Kurzrock R Spigel DR Burris HA Sweeney CJ. Atezolizumab treatment of tumors with high tumor mutational burden from MyPathway, a multicenter, open-label, phase IIa multiple basket study. Cancer Discovery. (2022) 12:654–69. doi: 10.1158/2159-8290.CD-21-0450 PMC9394388 34876409

[14] Girard N Bar J Garrido P Garassino MC McDonald F Mornex F. Treatment characteristics and real-world progression-free survival in patients with unresectable stage III NSCLC who received durvalumab after chemoradiotherapy: findings from the PACIFIC-R study. J Thorac Oncol. (2023) 18:181–93. doi: 10.1016/j.jtho.2022.10.003 36307040

[15] Kerr KM Lamb D. Actual growth rate and tumour cell proliferation in human pulmonary neoplasms. Br J Cancer. (1984) 50:343–49. doi: 10.1038/bjc.1984.181 PMC1976798 6087867

[16] Trott KR. Cell repopulation and overall treatment time. Int J Radiat Oncol Biol Phys. (1990) 19:1071–75. doi: 10.1016/0360-3016(90)90036-j 2211245

[17] De Ruysscher D Pijls-Johannesma M Bentzen SM Minken A Wanders R Lutgens L. Time between the first day of chemotherapy and the last day of chest radiation is the most important predictor of survival in limited-disease small-cell lung cancer. J Clin Oncol. (2006) 24:1057–63. doi: 10.1200/JCO.2005.02.9793 16505424

[18] Said BI Geng Y Badiyan SN Bang A Bezjak A Chua K. Accelerated hypofractionated radiotherapy for locally advanced NSCLC: A systematic review from the international association for the study of lung cancer advanced radiation technology subcommittee. J Thorac Oncol. (2024) 20(1):39–51. doi: 10.1016/j.jtho.2024.09.1437 39349294 PMC11724749

[19] Robinson SD Tahir BA Absalom K Lankathilake A Das T Lee C. Radical accelerated radiotherapy for non-small cell lung cancer (NSCLC): A 5-year retrospective review of two dose fractionation schedules. Radiother Oncol. (2020) 143:37–43. doi: 10.1016/j.radonc.2019.08.025 31563408

[20] Agolli L Valeriani M Bracci S Nicosia L DE Sanctis V Enrici RM. Hypofractionated image-guided radiation therapy (3Gy/fraction) in patients affected by inoperable advanced-stage non-small cell lung cancer after long-term follow-up. Anticancer Res. (2015) 35:5693–700. doi: 10.1016/S0167-8140(15)41167-3 26408745

[21] Huang RX Zhou PK. DNA damage response signaling pathways and targets for radiotherapy sensitization in cancer. Signal Transduct Target Ther. (2020) 5:60. doi: 10.1038/s41392-020-0150-x 32355263 PMC7192953

[22] Hughes JR Parsons JL. FLASH radiotherapy: current knowledge and future insights using proton-beam therapy. Int J Mol Sci. (2020) 21(18):6492. doi: 10.3390/ijms21186492 32899466 PMC7556020

[23] Ochoa DOM Bourhis J Irving M Coukos G Herrera FG. High versus low dose irradiation for tumor immune reprogramming. Curr Opin Biotechnol. (2020) 65:268–83. doi: 10.1016/j.copbio.2020.08.001 32882511

[24] Demaria S Guha C Schoenfeld J Morris Z Monjazeb A Sikora A. Radiation dose and fraction in immunotherapy: one-size regimen does not fit all settings, so how does one choose? J Immunother Cancer. (2021) 9(4):e002038. doi: 10.1136/jitc-2020-002038 33827904 PMC8031689

[25] Rodriguez-Ruiz ME Rodriguez I Leaman O Lopez-Campos F Montero A Conde AJ. Immune mechanisms mediating abscopal effects in radioimmunotherapy. Pharmacol Ther. (2019) 196:195–203. doi: 10.1016/j.pharmthera.2018.12.002 30529041

[26] Lynch C Pitroda SP Weichselbaum RR. Radiotherapy, immunity, and immune checkpoint inhibitors. Lancet Oncol. (2024) 25:e352–62. doi: 10.1016/S1470-2045(24)00075-5 39089313

[27] Bestvina CM Pointer KB Karrison T Al-Hallaq H Hoffman PC Jelinek MJ. A phase 1 trial of concurrent or sequential ipilimumab, nivolumab, and stereotactic body radiotherapy in patients with stage IV NSCLC study. J Thorac Oncol. (2022) 17:130–40. doi: 10.1016/j.jtho.2021.08.019 34500113

[28] Chang JY Lin SH Dong W Liao Z Gandhi SJ Gay CM. Stereotactic ablative radiotherapy with or without immunotherapy for early-stage or isolated lung parenchymal recurrent node-negative non-small-cell lung cancer: an open-label, randomised, phase 2 trial. Lancet. (2023) 402:871–81. doi: 10.1016/S0140-6736(23)01384-3 PMC10529504 37478883

